# Single-Crystal Y_2_O_3_ Epitaxially on GaAs(001) and (111) Using Atomic Layer Deposition

**DOI:** 10.3390/ma8105364

**Published:** 2015-10-19

**Authors:** Y. H. Lin, C. K. Cheng, K. H. Chen, C. H. Fu, T. W. Chang, C. H. Hsu, J. Kwo, M. Hong

**Affiliations:** 1 Department of Physics, National Taiwan University, Taipei 10617, Taiwan; f01222018@ntu.edu.tw (Y.H.L.); r01222061@ntu.edu.tw (K.H.C.); r01222066@ntu.edu.tw (C.H.F.); 2 Graduate Institute of Applied Physics, National Taiwan University, Taipei 10617, Taiwan; b98201018@ntu.edu.tw (C.K.C.); b99202021@ntu.edu.tw (T.W.C.); 3 National Synchrotron Radiation Research Center, Hsinchu 30076, Taiwan; 4 Department of Physics, National Tsing Hua University, Hsinchu 30013, Taiwan

**Keywords:** atomic layer deposition, single crystal, epitaxial, molecular beam epitaxy, (001) and (111) orientations, interfacial trap density

## Abstract

Single-crystal atomic-layer-deposited (ALD) Y_2_O_3_ films 2 nm thick were epitaxially grown on molecular beam epitaxy (MBE) GaAs(001)-4 × 6 and GaAs(111)A-2 × 2 reconstructed surfaces. The in-plane epitaxy between the ALD-oxide films and GaAs was observed using *in-situ* reflection high-energy electron diffraction in our uniquely designed MBE/ALD multi-chamber system. More detailed studies on the crystallography of the hetero-structures were carried out using high-resolution synchrotron radiation X-ray diffraction. When deposited on GaAs(001), the Y_2_O_3_ films are of a cubic phase and have (110) as the film normal, with the orientation relationship being determined: Y_2_O_3_$\left(110\right)\left[001\right]\left[\bar{1}10\right]$//GaAs$\left(001\right)\left[110\right]\left[1\bar{1}0\right]$. On GaAs(111)A, the Y_2_O_3_ films are also of a cubic phase with (111) as the film normal, having the orientation relationship of Y_2_O_3_$\left(111\right)\left[2\bar{1}\bar{1}\right]\left[01\bar{1}\right]$//GaAs$\left(111\right)\left[\bar{2}11\right]\left[0\bar{1}1\right]$. The relevant orientation for the present/future integrated circuit platform is (001). The ALD-Y_2_O_3_/GaAs(001)-4 × 6 has shown excellent electrical properties. These include small frequency dispersion in the capacitance-voltage (*CV*) curves at accumulation of ~7% and ~14% for the respective p- and n-type samples with the measured frequencies of 1 MHz to 100 Hz. The interfacial trap density (*D_it_*) is low of $\sim 10^{12}$ cm^−2^eV^−1^ as extracted from measured quasi-static *CV*s. The frequency dispersion at accumulation and the *D_it_* are the lowest ever achieved among all the ALD-oxides on GaAs(001).

## 1. Introduction

Single crystal rare earth (RE) oxides have been epitaxially grown on GaAs [1,2,3], Si [4,5,6,7], and GaN [8,9] using ultra-high vacuum (UHV) e-beam evaporation in a growth mode of molecular beam epitaxy (MBE) and atomic layer deposition (ALD). Among various high κ dielectrics in amorphous and single-crystal forms to passivate GaAs(001), MBE-grown Gd_2_O_3_-based RE-oxides have given low interfacial trap densities (*D_it_*) [10], thermal stability at high temperatures [11], and the first demonstration of inversion-channel enhancement-mode GaAs metal-oxide-semiconductor field-effect-transistor (MOSFET) [12,13].

ALD, on the other hand, has been widely employed in depositing high κ’s on Si in the semiconductor industry since the 45 nm node complementary MOS (CMOS), due to its advantages of conformal coverage and its self-limiting nature. The conformability is particularly critical for non-planar devices. Intensive research efforts using the two most common ALD oxides of Al_2_O_3_ and HfO_2_ to passivate GaAs(001) have, therefore, been taken to reduce *D_it_*’s [14,15,16,17,18,19,20,21,22]. Surface treatments including employing N_2_ and insertion of interfacial passivation layers were used prior to ALD [23,24]. However, very strong disparity in the measured capacitance-voltage curves (*CV*s) between n- and p-GaAs(001) using these two ALD oxides has been observed, with the n-type ones showing very large frequency dispersion at accumulation. The *D_it_* values in the GaAs band gap were a high >10^13^ cm^−2^eV^−1^, notably with a large *D_it_* peak at the mid-gap. The research efforts using ALD-Al_2_O_3_ and HfO_2_ have not yet produced low *D_it_*’s. In contrast, SiO_2_/Si has given very small frequency dispersion in the *CV*s for both n- and p-Si(001), and low *D_it_*’s. Also, a U-shape *D_it_* curve within the Si band-gap is attained without a peak at mid-gap.

Recently, ALD-LaLuO_3_ and -La_2−x_Y_x_O_3_ on GaAs(111) were found to be of single crystal [3], showing *CV*s with a small dispersion at accumulation for both p- and n-GaAs(111) substrates and a low *D_it_* [3], similar to what has been achieved earlier using the MBE-RE oxides on GaAs(001) [1,10,13]. A small lattice mismatch between the ALD-RE oxides and GaAs was speculated to lead to good oxide growth and interfacial electrical performances [3]. However, these ALD-oxides were polycrystalline when being deposited on GaAs(001) and amorphous on Si(111) [25], resulting in poor electrical performances. These were similar to what was observed in ALD-Al_2_O_3_ and -HfO_2_ on GaAs(001) [14,15,16,17,18,19,20,21]; namely large frequency dispersion in the *CV*s and a high *D_it_*. Large *D_it_* peak at the mid-gap of GaAs is always shown in ALD-oxides/GaAs [17], which retards the Fermi level moving across the energy gap.

High carrier (electron or hole) mobility semiconductors of GaAs [13,14], InGaAs [26,27,28,29], GaSb [30,31], and Ge [32,33,34] are now the leading candidates for replacing Si channel to enable high performance inversion-channel MOSFETs with low power consumption. These semiconductors have to be integrated onto Si with (001) as the substrate normal, which is the platform for the present integrated circuit (IC). The growth of these semiconductors on Si(001) results in (001), not (111) orientation.

The question is whether a small lattice mismatch is mandated for achieving epitaxial growth of RE-oxides, which are more ionic, on a more covalent GaAs. A second question is whether ALD-RE oxides can be epitaxially grown on GaAs(001) and at the same time give a low *D_it_*, which was achieved using MBE-RE oxides [1,10,13].

In our very recent work, ALD-Y_2_O_3_ 2.3 nm thick directly deposited on GaAs(001) was found to be epitaxial, as readily observed using *in-situ* reflection high energy electron diffraction (RHEED) [35]. The synchrotron radiation X-ray diffraction (XRD) radial scans along surface normal, rocking curves, azimuthal cone scans across off-specular reflections and crystal truncation rod measurements of ALD-Y_2_O_3_/GaAs(001) have established that the thin Y_2_O_3_ is a cubic single crystal with its (110) planes parallel with GaAs(001) surface and the in-plane directions [001] and [$\bar{1}$10] parallel with the [110] and [1$\bar{1}$0] of GaAs (001) [35]. The crystallographic structure of ALD-Y_2_O_3_ 2.3 nm thick is similar to that of single crystal MBE-Gd_2_O_3_ epitaxially grown on GaAs(001) which is the key for attaining a low *D_it_* for n-GaAs(001) [1,2,10,13].

From the RHEED and XRD studies, we found that the 2 nm thick ALD-Y_2_O_3_ grown on GaAs(111) is also a cubic phase but has (111) normal. It has the in-plane directions of [2$\bar{1}\bar{1}$] and [01$\bar{1}$] parallel with the [$\bar{2}$11] and [0$\bar{1}$1] of GaAs(111), respectively. A better two-dimensional (2-D) growth of Y_2_O_3_ film on GaAs(111)A has been observed.

The MOS capacitors (MOSCAPs) of the single crystal ALD-Y_2_O_3_/GaAs(001)-4 × 6 have exhibited very small frequency dispersion of the measured capacitance-voltage (CVs) curves at accumulation and *D_it_* values of ~10^12^ cm^−2^eV^−1^, having no peak at mid-gap in its distribution within the energy band gap.

## 2. Results and Discussion

**Figure 1:**
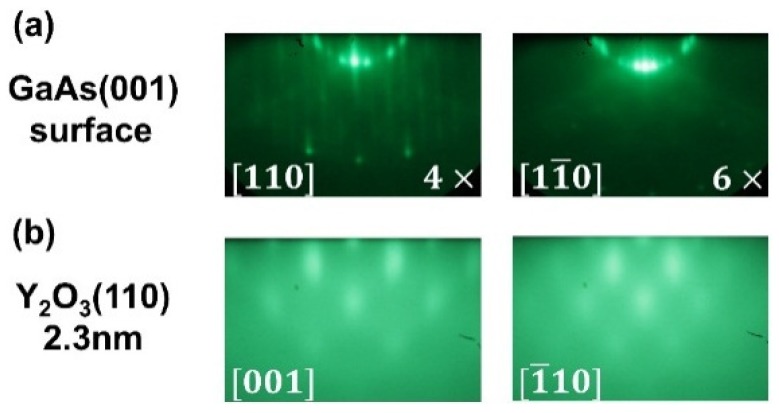
*In-situ* RHEED patterns of (**a**) freshly MBE grown GaAs(001)-4 × 6; and (**b**) subsequent growth of ALD-Y_2_O_3_(110) films 2.3 nm. The zone axes for the growth on GaAs(001) from the left to the right are GaAs[110], [1$\bar{1}$0], and ALD-Y_2_O_3_[001] and [$\bar{1}$10], respectively.

Figure 1a shows the GaAs(001)-4 × 6 reconstructed RHEED patterns along [110] and [1$\bar{1}$0] directions. The GaAs RHEED patterns and their in-plane symmetry changed to those of cubic Y_2_O_3_(110) after the deposition of the ALD-Y_2_O_3_ ~1 nm thick. Figure 1b shows the pattern for the oxide film 2.3 nm thick along the Y_2_O_3_ in-plane [001] and [$\bar{1}$10] directions. Note that the bulk lattice constant of bixbyite Y_2_O_3_, a_Y_2_O_3__ = 1.060 nm, is approximately twice that of GaAs, a_GaAs_ = 0.565 nm. Thus, there exists a large lattice mismatch, $\left({\text{d}}_{{\text{Y}}_{2}{\text{O}}_{3}}/2-{\text{d}}_{\text{GaAs}}\right)/{\text{d}}_{\text{GaAs}}\;$of 6.2% and 32.7% along the in-plane [001] and [$\bar{1}$10] directions of Y_2_O_3_(110), respectively, where d stands for the lattice spacing along the commonly aligned directions.

**Figure 2:**
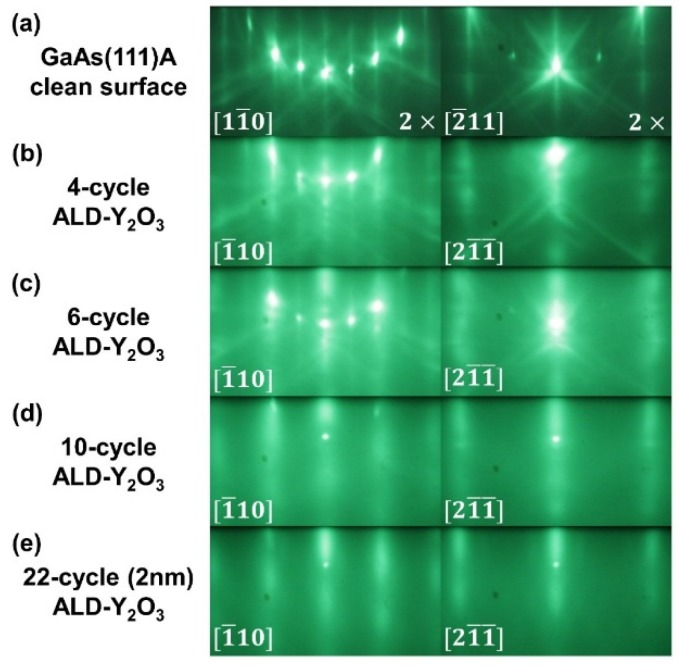
*In-situ* RHEED patterns of (**a**) freshly MBE grown GaAs(111)A-2 × 2, and subsequent growth of ALD-Y_2_O_3_(111) films (**b**) 4-cycle ALD-Y_2_O_3_; (**c**) 6-cycle; (**d**) 10-cycle; and (**e**) 22-cycle (2 nm). The zone axes for left panel are, respectively, GaAs[1$\bar{1}$0] and ALD-Y_2_O_3_[$\bar{1}$10], and for the right panel are GaAs[$\bar{2}$11] and ALD-Y_2_O_3_[2$\bar{1}\bar{1}$], respectively.

Figure 2a shows the GaAs(111)A-2 × 2 reconstructed RHEED patterns along the in-plane [$\bar{2}11$] and [1$\bar{1}$0] directions. The sharp, bright, and narrow RHEED streaks of GaAs(111)A with obvious Kikuchi arcs indicate the excellent crystallinity of GaAs growth with a well-ordered structure. Different from the epitaxial growth on GaAs(001), the co-existence of the RHEED patterns from both GaAs and the oxide was observed for the very first few cycles (2–6 cycles) of ALD-Y_2_O_3_ on GaAs (111)A as shown in Figure 2b,c of 4- and 6-cycle deposition. The RHEED streaks were fattened with the deposition of thin ALD-Y_2_O_3_ films, indicating that the films have the same in-plane symmetry, but with less crystallographic order and different lattice spacing. The patterns of GaAs were not observable at the 10-cycle film deposition as shown in Figure 2d, which are streakier than the ALD-Y_2_O_3_ with similar thickness on GaAs(001). Now comparing the RHEED patterns of the 22-cycle (2 nm thick) ALD-Y_2_O_3_ on GaAs(111) (Figure 2e) with those of ALD-Y_2_O_3_ with similar thickness (2.3 nm thick) on GaAs(001) (Figure 1b), the latter shows a sausage-like pattern with a 2× reconstruction along the in-plane [$\bar{1}$10] (Figure 1b).

The large lattice mismatch and different bonding of the deposited oxide and semiconductor substrate, namely ionic *versus* more covalent bonds between Y_2_O_3_ and GaAs, has not prevented the epitaxial growth of ALD-Y_2_O_3_ on both GaAs(001) and (111). One also observed that the strained pseudomorphic growth did not occur even for very thin thicknesses such as 1 nm, which already exhibited Y_2_O_3_(110) as surface normal to the underlying GaAs(001).

The observation of the ALD epitaxial growth was made possible using our unique setup of connecting MBE chamber whose vacuum is maintained below 10^−10^ Torr, while the pressure in the ALD reactor is in the order of a few Torr during deposition. After the ALD, the reactor was pumped down to 10^−9^ Torr prior to the sample being transferred to the MBE chamber equipped with RHEED via the UHV modules.

**Figure 3:**
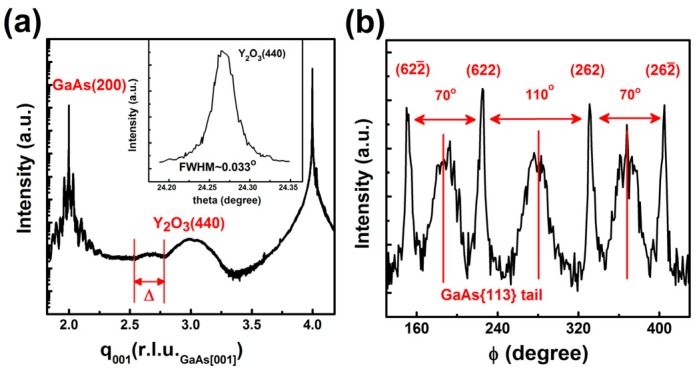
XRD (**a**) radial scans along the surface normal of ALD-Y_2_O_3_ film 2.3 nm thick on GaAs(001)-4 × 6 reconstructed surface; with (**b**) an azimuthal φ scan across the {622} reflections of ALD-Y_2_O_3_ film 5 nm thick on GaAs(001)-4 × 6. The inset in (**a**) is the θ-rocking curve across the Y_2_O_3_(440) reflection.

Synchrotron radiation source gives a high sensitivity to X-ray diffraction in studying very thin films with thickness in the range of a few nano-meters. We will start with the crystallographic structures of ALD-Y_2_O_3_ film 2.3 nm thick on GaAs(001)-4 × 6, and then move to the same oxide of a similar thickness on a different orientation of (111). The former structure was studied earlier [35]. The XRD radial scan along the surface normal of the former hetero-structure is shown in Figure 3a, in which the location of the broad peak appearing at the scattering vector along the surface normal q_001_ ~ 3.006 r.l.u._GaAs[001]_, reciprocal lattice unit of GaAs along the [001] direction with a value of 2π/a_GaAs_ Å^−1^, was very close to that of the (440) reflection of cubic Y_2_O_3_. Furthermore, no other peak except the GaAs reflections was observed in the radial scan. These observations indicated that the Y_2_O_3_ film had a cubic structure and was (110) oriented. From the periodicity, Δ~0.247 r.l.u._GaAs[001]_ shown in Figure 3a, of the interference fringe near the Y_2_O_3_(440) diffraction peak, the film thickness was estimated to be ~2.3 nm. In the region near the GaAs(002) reflection, the satellites and oscillation fringes were caused by the interference of the underlying AlGaAs/GaAs superlattice designed for blocking the diffusion of structural defects.

**Figure 4:**
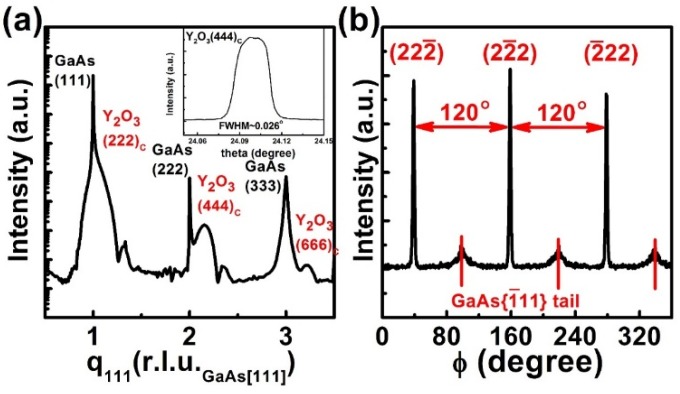
XRD (**a**) radial scans along the surface normal of ALD-Y_2_O_3_ films 2 nm thick on GaAs(111)A-2 × 2 reconstructed surface. The inset shows the θ-rocking curve across the Y_2_O_3_(444) reflection; (**b**) Azimuthal φ scan across the {$\bar{2}$22} reflections of the ALD-Y_2_O_3_ film 2 nm thick on GaAs(111)A-2 × 2 reconstructed surface.

As to the growth of ALD-Y_2_O_3_(111) on GaAs(111)A, the Y_2_O_3_ layer also has a cubic structure but with its (111) planes parallel with the GaAs(111) surface. XRD radial scan (theta *versus* two-theta scan) along surface normal of the 2.0 nm thick ALD-Y_2_O_3_ is displayed in Figure 4a. The broad peaks appearing at high q_111_ side of each intense GaAs reflection are attributed to the Y_2_O_3_ layer, where q_111_ is the scattering vector along GaAs[111] direction. Their peak positions, 1.077, 2.154, and 3.218 r.l.u._GaAs[111]_, reciprocal lattice unit of GaAs along [111] with a value of $\sqrt{3}\cdot$2π/a_GaAs_ Å^−1^, approaching those of the (222), (444), and (666) reflections of bulk cubic phase Y_2_O_3_, respectively. The tails of the nearby GaAs reflections make the accurate determination of the peak positions difficult. This explains why the measured positions of the Y_2_O_3_ peaks are not exactly proportional to their Miller indices. The film thicknesses were estimated to be ~2 nm from the periodicity of the thickness fringes near the Y_2_O_3_(222) and (444) reflections. 

Previous study on MBE-Y_2_O_3_ grown on GaN(0001) showed that Y_2_O_3_ can exist in hexagonal phase as the film thickness ≤3 nm and its crystalline structure resembles that of the cubic phase in many aspects [36]. It is risky to identify the phase by the specular reflections alone and thus essential to examine the positions of the off-normal reflections.

For the Y_2_O_3_ film grown on GaAs(001), the azimuthal φ scan across cubic Y_2_O_3_(622) reflection shows four sharp peaks (Figure 3b). The larger angular separation ~110° agreed well with the calculated 109.5° between the (622) and (262) pair and between the (62$\bar{2}$) and (26$\bar{2}$) pair. On the other hand, the smaller separation 70° matches the angular spacing between the (622) and ($62\bar{2}$) pair and between the (262) and ($26\bar{2}$) pair. The four evenly spaced broad peaks were the tails of the GaAs{113} reflections. The epitaxial relationship between the Y_2_O_3_ film and GaAs substrate deduced from the relative position of the reflections is Y_2_O_3_(110)[$\bar{1}$10]/GaAs(001)[1$\bar{1}$0] and only one rotational domain exists. With the determined orientation, we estimated that the lattice constant of Y_2_O_3_ had a small, ~0.3%, dilation along the surface normal. More data is required to evaluate the bi-axial lateral strains.

Azimuthal φ scans across the ($\bar{2}22$) reflection of cubic Y_2_O_3_ on GaAs(111) are depicted in Figure 4b. Three evenly spaced sharp peaks yield the characteristic 3-fold symmetry along cubic [111] axis. The weak broad peaks between the intense peaks originate from the tail of the nearby GaAs{$\bar{1}$11} reflections. The 60° offset between the two sets of reflections elucidates the B-type cube-on-cube growth, *i.e.*, Y_2_O_3_[$2\bar{1}\bar{1}$]//GaAs[$\bar{2}11$], consistent with the observed RHEED patterns, and there exists only one rotational variant. From the diffraction peak positions, we derived that the Y_2_O_3_(111) film is compressively strained by 0.6% along the growth direction and tensile strain by 0.9% laterally. The observed strain is much less than the calculated lattice mismatch, indicating a significant lattice relaxation, most probably through the generation of misfit dislocation at the interface.

The intensities were displayed in an arbitrary unit to illustrate the signals from both Y_2_O_3_ and GaAs. The intensity of the GaAs{$\bar{1}$11} reflections of the (111)-oriented GaAs in Figure 4b should not be compared directly with that of the {113} reflections of the (001)-oriented GaAs in Figure 3b. The much more intense GaAs(113) tail was resulted from the relatively weak Y_2_O_3_{622} reflections.

The full width at half maximum (FWHM) of the Y_2_O_3_(444) rocking curve was 0.026°. The narrow FWHM of the rocking curve indicates the excellent crystallinity of ALD-Y_2_O_3_ film on GaAs(111)A, which is better than the ALD-Y_2_O_3_ grown on GaAs(001), of which the (440) rocking curve width is 0.033°.

A small lattice mismatch between deposited films and the substrates underneath is usually preferred for the epitaxial growth [3,37]. Numerous examples on the hetero-epitaxial growth with large lattice mismatches have nevertheless been demonstrated. These have led to excellent crystallographic characteristics, interesting scientific discoveries, and very useful important technologies [1,2,38,39,40]. Here we demonstrate that, even with a large lattice mismatch, high-quality epitaxial Y_2_O_3_ films have been grown on GaAs(001) and (111)A by ALD and the hetero-structures exhibit impressive electrical characteristics.

**Figure 5:**
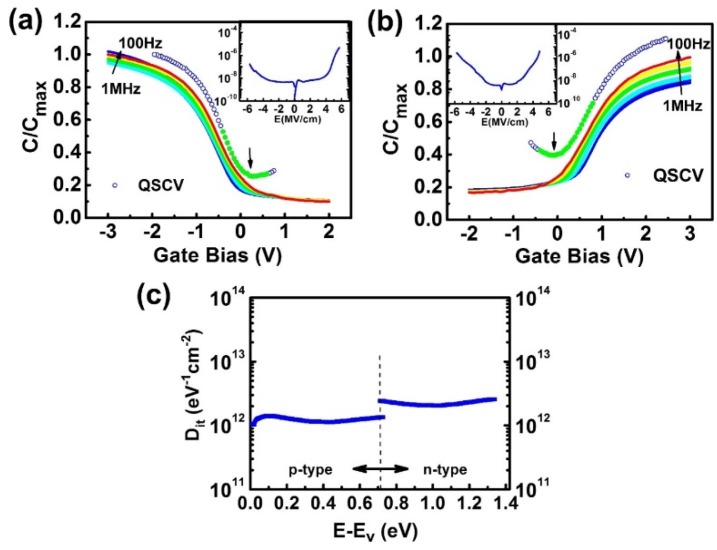
*CV*s of ALD-Al_2_O_3_/ALD-Y_2_O_3_(2.3 nm)/GaAs(001)-4 × 6 MOSCAPs for p-type GaAs (**a**) and for n-type GaAs (**b**) with insets showing their respective *JE* curves; and (**c**) showing *D_it_* distribution within GaAs band gap.

The systematic studies on the electrical performances of the ALD-Y_2_O_3_/p- and n-GaAs(001) are given in a separate publication [41]. Low frequency dispersion from 1 MHz to 100 Hz at accumulation in the *CV*s has been attained with ~7% and ~14% for p- and n-type GaAs(001)-4 × 6, respectively, as shown in Figure 5a,b. These are the record low values among all the ALD-Al_2_O_3_ and -HfO_2_ on GaAs(001), of which the frequency dispersion at accumulation region of *CV*s on n-type GaAs(001) is high. For example, the values were reported to be ~60%, ~40%, and ~23% in refs. [17,21,42], respectively. Note that the 23% was attained with frequency measured from 100 kHz to 100 Hz.

The current density-field (*JE*) characteristics (insets of Figure 5a,b) showed low leakage current densities <10^−8^ A/cm^2^ at ± 1 MV/cm for the MOSCAPs of ALD-Al_2_O_3_ (4 nm)/Y_2_O_3_/(2.3 nm)/p- and n-GaAs(001); the low leakage allows the reliable quasi-static *CV* measurements. Low *D_it_* values of (1–3) × 10^12^ cm^−2^eV^−1^, extracted from the quasi-static *CV*s [43,44], are shown in Figure 5c. Moreover, while the GaAs(001) MOSCAPs using other ALD-oxides always showed large *D_it_* peak values at the mid-gap [17,20,22], the *D_it_* spectrum here showed a flat distribution across whole bandgap. Thus, Fermi level at the Y_2_O_3_/GaAs interface can be moved effectively across the bandgap of GaAs with applying gate voltage, the key to high performance device. 

## 3. Experimental Section

MBE was employed for the epi-layer growth of GaAs(001) and (111), and ALD for the high κ Y_2_O_3_ films. Both the MBE chamber and the ALD reactor are in a multi-chamber ultrahigh vacuum (UHV) growth/analysis system, which also includes an arsenic-free metal/oxide MBE chamber, and two analysis chambers of scanning tunneling microscopy (STM) and X-ray photoelectron spectroscopy (XPS) [42]. The aforementioned chambers are connected via transfer modules, which maintained 10^−10^ Torr to ensure maintenance of the pristine sample surfaces free of contamination. After the MBE growth of the GaAs epi-layers 30–50 nm thick with Si and Be as the n- and p-dopants, respectively, the samples were transferred *in-situ* under UHV to an As-free MBE chamber to attain GaAs(001)-4 × 6 and (111)A-2 × 2 surface reconstructions by annealing the samples to 550 °C; these were monitored by *in-situ* RHEED and were confirmed by low-energy electron diffraction (LEED) in the photoemission chamber at the nearby National Synchrotron Radiation Research Center (NSRRC) to which the samples were transferred using a battery-powered portable UHV chamber (with a vacuum of 10^−^^10^ Torr) [45]. 

Also, no native oxides were detected, as examined using *in-situ* XPS and synchrotron radiation photoemission under the same transfer procedure [46]. The freshly MBE grown GaAs(001)-4 × 6 and GaAs(111)A-2 × 2 samples were *in-situ* transferred to the ALD reactor under UHV. The ALD-Y_2_O_3_ process was carried out at a substrate temperature of 270 °C with the precursors of tris(ethylcyclopentadienyl) yttrium and water [47]. The ALD-growth starts with first pulse of tris(ethylcyclopentadienyl) yttrium. The oxide growth was monitored by *in situ* RHEED. A 5 nm thick ALD-Al_2_O_3_ was deposited on the Y_2_O_3_ as a cap layer for protection. Structural characterization by XRD was conducted with an 8-circle diffractometer in NSRRC, Taiwan. ALD-Al_2_O_3_/ALD-Y_2_O_3_/n- and p-GaAs(001)-4 × 6 MOS capacitors (MOS CAPs) were used to measure electrical characteristics, with e-beam evaporated Ni as the gate metals. The circular pattern was formed through a shadow mask with a diameter of 100 μm. Capacitance-voltage (*C-V*) characteristics were measured at room temperature using an Agilent 4284 LCR meter. Quasi-static *CV* (*QSCV*) and electrical leakage current density-field (*JE*) measurements were performed in dark at room temperature using an Agilent 4156C. The *D_it_*’s were calculated from the *QSCV* data.

## 4. Conclusions

Previously, single crystal rare earth oxide films were grown on GaAs(001) and GaAs(111)A using molecular beam epitaxy (MBE) and on GaAs(111) using ALD. Here, we have used ALD to grow single-crystal Y_2_O_3_ epitaxially on both GaAs(001) and (111) reconstructed surfaces despite a large lattice mismatch. To our knowledge, it has not been reported that single crystal ALD oxides were grown on GaAs(001). Equally important, excellent crystallinity was demonstrated on ALD-Y_2_O_3_(2 nm)/GaAs(111) with a better two-dimensional growth of Y_2_O_3_ film. Advancing from what was achieved using MBE-prepared single crystal RE oxides on effectively passivating various semiconductors with a normal of (001), in this work, we have used single crystal ALD-Y_2_O_3_/GaAs(001) to attain small frequency dispersion in the *CV*s, low *D_it_* values with a flat distribution having no peak near GaAs mid-gap. These excellent electrical results have not been achieved using other ALD oxides such as Al_2_O_3_ and HfO_2_. Note that the lattice mismatches in ALD-Y_2_O_3_/GaAs(001) are larger than those in ALD-Y_2_O_3_/GaAs(111).
